# Influence of Epinastine Hydrochloride, an H_1_-Receptor Antagonist, on the Function of Mite Allergen-Pulsed Murine Bone Marrow-Derived Dendritic Cells In Vitro and In Vivo

**DOI:** 10.1155/2009/738038

**Published:** 2009-04-09

**Authors:** Ken-Zaburo Oshima, Kazuhito Asano, Ken-Ichi Kanai, Miyuki Suzuki, Harumi Suzaki

**Affiliations:** ^1^Department of Otolaryngology, School of Medicine, Showa University, Tokyo 142-8555, Japan; ^2^Division of Physiology, School of Nursing and Rehabilitation Sciences, Showa University, Yokohama 226-8555, Japan

## Abstract

There is established concept that dendritic cells (DCs) play essential roles in the development of allergic immune responses. However, the influence of H_1_ receptor antagonists on DC functions is not well defined. The aim of the present study was to examine the effect of epinastine hydrochloride (EP), the most notable histamine H_1_ receptor antagonists in Japan, on *Dermatophagoides farinae (Der f)*-pulsed mouse bone marrow-derived DCs in vitro and in vivo. EP at more than 25 ng/mL could significantly inhibit the production of IL-6, TNF-*α* and IL-10 from *Der f*-pulsed DCs, which was increased by *Der f* challenge in vitro. On the other hand, EP increased the ability of *Der f*-pulsed DCs to produce IL-12. Intranasal instillation of *Der f*-pulsed DCs resulted in nasal eosinophilia associated with a significant increase in IL-5 levels in nasal lavage fluids. *Der f*-pulsed and EP-treated DCs significantly inhibited nasal eosinophila and reduced IL-5. These results indicate that EP inhibits the development of Th2 immune responses through the modulation of DC functions and results in favorable modification of clinical status of allergic diseases.

## 1. Introduction

Allergic rhinitis is an inflammation of the nasal passages, usually associated with watery nasal discharge, sneezing and itching of the nose. These clinical symptoms are also well accepted to be mediated by vasoactive substances, arachidonic acid metabolites, cytokines and chemokines secreted from inflammatory cells, including eosinophils, mast cells, and T cells [1]. The initial response in the development of allergic inflammation is an allergen, such as mite and pollen, being presented to the nasal mucosa. This allergen is then recognized as an antigen by antigen-presenting cells (APCs) and presented to plasma cells. These cells subsequently produce IgE and attach themselves to mast cells and eosinophils awaiting re-exposure. Upon re-exposure, an antigen-antibody complex is formed and activate inflammatory cells to secrete several factors responsible for allergic immune responses [2].

The goal of management of allergic immune responses, including allergic rhinitis is to reduce the clinical symptoms caused by the inflammation of affected tissues. Avoidance of the allergens or minimization of contact with them is the best treatment, but some relief may be found with the pharmacological medications. Pharmacotherapy available for allergic rhinitis is vast. Although several studies have demonstrated that corticosteroids are the most effective agents in the treatment of allergic airway diseases, including allergic rhinitis, the use of this class of drugs may be limited because of concerns over unwanted side effects [3]. In contrast, antihistamines are commonly used as the first-line treatment for allergic rhinitis and successful results are reported [3–5]. Although primal target of antihistamines is the histamine H_1_ receptor, these drugs act as inhibitors of the synthesis and release of chemical mediators and inflammatory cytokines from eosinophils and mast cells following immunological and nonimmunological stimulations [6, 7]. Antihistamines also reported to be able to suppress the ability of leukocytes to produce inflammatory cytokines and chemokines in response to inflammatory stimulations [8, 9]. Furthermore, our previous works clearly showed the suppressive activity of antihistamines, especially fexofenadine hydrochloride, on nasal fibroblasts to produce inflammatory mediators, such as nitric oxide and matrix metalloproteinases induced by inflammatory stimulation [10, 11]. These reports strongly suggest that antihistamines modulate the function of cells, which are responsible for the development of allergic inflammation, and results in favorable modification of the allergic disease state or conditions. On the other hand, there is established concept that dendritic cells (DCs), the most potent APC in airways, play essential roles in the development of allergic immune responses through the production of immunomodulatory cytokines and expression of costimulatory molecules on their cell surface [12–14]. A number of studies reported the effects of several types of agents, cysteinyl leukotriens receptor antagonists [13, 15], *β*2-adrenargic receptor antagonists [16], and corticosteroids [13, 16], which are used for the treatment of airway inflammatory diseases, on DC functions, while a few examined the effects of antihistamines on DCs.

In the present study, we examined the influence of epinastine hydrochloride (EP), the most notable H_1_ receptor antagonist in Japan, on the functions of DCs using mite allergen-pulsed murine bone marrow-derived DCs in vitro and in vivo.

## 2. Materials and Methods

### 2.1. Mice

Specific pathogen-free female BALB/c mice, 7 weeks old, were purchased from Charles River Japan Inc., (Kanagawa, Japan) and maintained in our animal facilities at 25 ± 2°C, 55 ± 5% humidity and 12-hour light/dark cycle. Each experimental and control group consisted of five mice. All animal experimental procedures were approved by the Animal Care and Use Committee of Showa University.

### 2.2. Reagents

EP was kindly donated from Nippon Boehringer Ingelheim Co., Ltd. (Tokyo, Japan) as a preservative-free pure powder. EP was dissolved in RPMI-1640 medium (Sigma Aldrich, Mo, USA) supplemented with 10% heat-inactivated fetal calf serum (RPMI-FCS) at a concentration of 1.0 mg/mL, sterilized by passing through a 0.2 *μ*m filter and stored as a stock solution at 4°C until used. Mite extract *Dermatophagoides farinae* (*Der f *) was obtained from Cosmo Bio Co., Ltd. (Tokyo, Japan). Contamination of lipopolysaccharide (LPS) in the *Der f* preparations was <0.96 EU/mg *Der f* as assessed by Limulus amebocyte lysate test (Sigma Aldrich). Recombinant mouse granulocyte/macrophage colony stimulating factor (rmGM-CSF), preservative free, was purchased from R&D Systems Inc. (Minn, USA).

### 2.3. Induction of DCs

Murine bone marrow-derived DCs were induced as described previously [17]. Briefly, femurs and tibiae of BALB/c mice (10 mice) were removed and purified the surrounding muscle tissue. Both ends were cut and the marrow flushed with phosphate-buffered saline (PBS). After washing five times with PBS containing 500 U/mL penicillin, 500 *μ*g/mL streptomycin, and 10 *μ*g/mL amphotericin B, bone marrow cells were suspended in RPMI-FCS at a concentration of 2 × 10^6^ cells/mL and cultured in 100 mm culture plates in a final volume of 10.0 mL. At day 3, another 10.0 mL of RPMI-FCS containing 20.0 ng/mL rmGM-CSF was added to the plates. On day 6, half of the culture medium was changed with fresh medium containing 5.0 ng/mL rmGM-CSF.

### 2.4. Preparation of Antigen-Pulsed and EP-Treated DC

DCs were collected on day 8, washed once with RPMI-FCS, resuspended at 2 × 10^6^ cells/mL in RPMI-FCS that contained 5.0 ng/mL rmGM-CSF. These cells were then introduced into 24-well culture plates in triplicate that contained various concentrations (0, 20, 25, 30, and 35 ng/mL) of EP in a final volume of 2.0 mL. After 2 hour, *Der f* was added to cell cultures to give a final concentration of 100 *μ*g/mL. Cell-free culture supernatants collected after 24-hour incubation were stored at −80°C until used. To prepare *Der f*-pulsed and EP-treated DCs for in vivo use, DCs collected on day 8 were cultured with 25.0 ng/mL EP in a final volume of 10.0 mL. After 2 hours, 100 *μ*g/mL *Der f* was added to cell cultures, and incubated for a further 24 hours. The cells were then collected, washed three times, and resuspended in PBS at concentration of 2 × 10^7^ cells/mL and used for in vivo experiments.

### 2.5. Immunization with *Der f*-Pulsed DCs and Exposure to Allergen In Vivo

Naïve BALB/c mice were lightly anesthetized with ether and divided into six groups (five mice/group). To prepare non-DC instilled control, mice were received intranasally with 50 *μ*g of *Der f* once daily for 5 consecutive days in a volume of 50 *μ*L. *Der f* nonpulsed control DCs and *Der f*-pulsed DCs (1 × 10^6^ cells/50 *μ*L) was applied at the tip of the nose with a micropipet and inhaled involuntarily [15, 16, 18]. These mice were then challenged intranasally with either PBS or 50 *μ*g of *Der f* (50 *μ*L, each) once daily for 5 consecutive days, which was started 10 days after cell instillation. The remaining one group of mice were intranasally instilled with *Der f*-pulsed DCs pretreated with 25 ng/mL EP and challenged with *Der f* in a similar manner.

### 2.6. Preparation of Nasal Cavity Lavage Fluid (NLF)

Mice were killed by intraperitoneal injection with 1.0 mL of 50 mg/mL sodium pentobarbital (Abbott Laboratories, Ill, USA) 24 hours after final *Der f* challenge [15, 16], and a midline incision was performed above the sternum. The trachea was exposed by blunt dissection and a 28 gauge plastic tube was inserted into trachea above the carina to nasal cavity. The both sides of nasal cavity were then simultaneously lavaged with 1.0 mL PBS. Aliquots of the nasal lavage fluid were then centrifuged at 3000 g at 4°C for 15 minutes, and the supernatants were collected and stored at −80°C until use. The pellets spended in 1 mL PBS were used for counting eosinophils and lymphocytes.

### 2.7. Assay for Cytokines

Concentrations of immunomodulatory cytokines, IL-12p40 and IL-10, and proinflammatory cytokines, IL-6 and TNF-*α* in DCs culture supernatants were measured using commercially available mouse cytokine ELISA assay kits (R&D Systems Inc.) according to the manufacturer's instructions. IL-5 and IFN-*γ* in NLF were also assayed by mouse cytokine ELISA assay kits (Pierce Biotechnology Inc., Ill, USA). The detectable minimum levels of these ELISA kits were 15 pg/mL for IL-12p40, 15.0 pg/mL for IL-10, 3.0 pg/mL for IL-6, 3.0 pg/mL for TNF-*α*, 5.0 pg/mL for IL-5, 10.0 pg/mL for IFN-*γ*, respectively.

### 2.8. Counting for Lymphocytes and Eosinophils in NLF

Total number of lymphocytes in NLF were counted with Turk's solution and hemocytometers. The number of eosinophils in NLF was examined using Hinkelman's solution and hemocytometers.

### 2.9. Statistical Analysis

Statistical difference were examined using analysis of variance (ANOVA), followed by Fisher's PLSD test. A *P*-value less than .05 denoted the presence of a statistically significant difference.

## 3. Results

### 3.1. Influence of EP on Cytokine Production from DCs In Vitro

**Figure 1:**
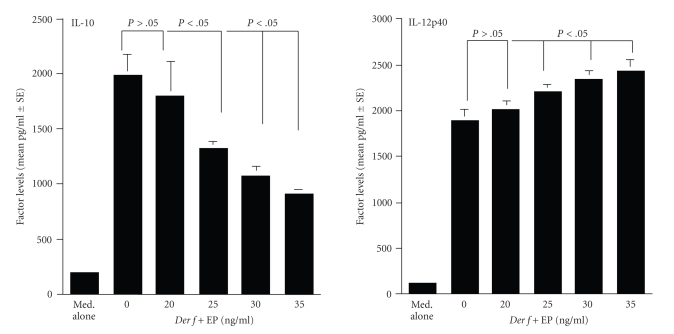
Influence of epinastine hydrochloride (EP) on immunomodulatory cytokine production from dendritic cells (DCs) stimulated with *Dermatophagoides farinae* (*Der f *) in vitro. DCs derived from bone marrow of BALB/c mice were stimulated with *Der f* in the presence of various concentrations of EP for 24 hours. IL-10 and IL12p40 levels in culture supernatants were examined by ELISA. The data are expressed as the mean pg/mL ± SE of triplicate cultures. This is one of two different experiments, which gave reproducible results.

**Figure 2:**
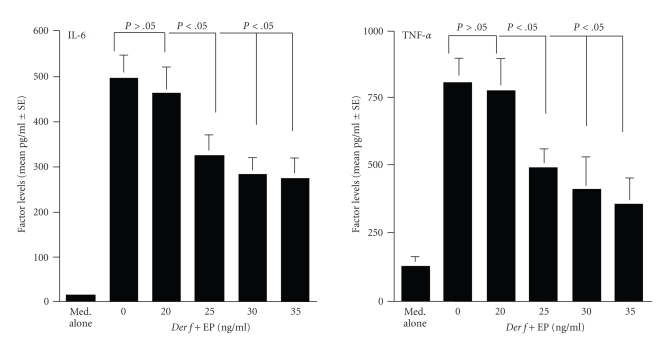
Influence of epinastine hydrochloride (EP) on proinflammatory cytokine production from dendritic cells (DCs) stimulated with *Dermatophagoides farinae* (*Der f *) in vitro. DCs derived from bone marrow of BALB/c mice were stimulated with *Der f* in the presence of various concentrations of EP for 24 hours. IL-6 and TNF-*α* levels in culture supernatants were examined by ELISA. The data are expressed as the mean pg/mL ± SE of triplicate cultures. This is one of two different experiments, which gave reproducible results.

The first experiments were undertaken to examine the influence of EP on cytokine production from DCs after *Der f* stimulation in vitro. As shown in Figure 1, DCs could produce much higher (*P* < .05) levels of IL-10 in response to *Der f* stimulation as compared with nonstimulated DCs. On the other hand, addition of EP into cell cultures dose dependently suppressed the ability of DCs to produce IL-10 (Figure 1). The minimum concentration of EP that caused significant suppression was 25 ng/mL (Figure 1). In contrast to the case of IL-10, IL-12p40 levels in culture supernatants was further increased by the treatment of DCs with EP, when EP at more that 25 ng/mL was added to cell cultures (Figure 1). We then examined the influence of EP on the production of IL-6 and TNF-*α* from DCs in response to *Der f* stimulation. As shown in Figure 2, treatment of DCs with EP at more than 25 ng/mL significantly suppressed the production of IL-6 and TNF-*α*, which were increased by *Der f* stimulation in vitro.

### 3.2. Influence of Intranasal Instillation of DCs on the Appearance of Inflammatory Cells in NLF

**Figure 3:**
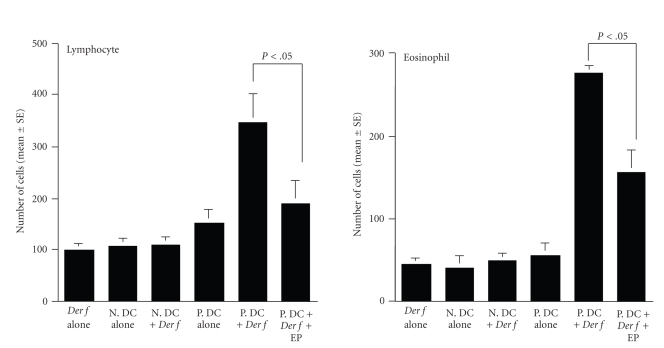
Influence of nasal antigenic challenge on inflammatory cell appearance in nasal lavage fluid obtained from mice instilled nasally with *Dermatophagoides farinae* (*Der f *)-pulsed dendritic cells. BALB/c mice were instilled nasally with *Der f*-pulsed and 25 ng/mL epinastine hydrochloride (EP)-treated DCs. Nasal lavage was performed 24 hours after the final *Der f* allergen exposure. The data are expressed as the mean number of cells ± SE of five mice. This is one of two different experiments, which gave reproducible results. *Der f* alone: *Der f* allergen exposure alone; N.DC alone: nonpulsed DC instilled; N.DC + *Der f* : nonpulsed DC instilled and *Der f* allergen exposure; P. DC alone: *Der f*-pulsed DC instilled; P. DC + *Der f* : *Der f*-pulsed DC and *Der f* allergen exposure; P. DC + *Der f* + EP: *Der f*-pulsed, EP-treated DC and *Der f* exposure.

The second experiments were designed to examine the influence of intranasal instillation of DCs on inflammatory cell appearance in nasal wall. As shown in Figure 3, intranasal instillation of *Der f*, nonpulsed DCs, nonpulsed DCs and challenged *Der f*, and pulsed DCs could not cause the apparent changes in inflammatory cell appearance in nasal wall: NLF obtained from these groups of mice contained similar numbers (not significant; *P* < .05) of both eosinophils and lymphocytes. On the other hand, NLF obtained from mice instilled with *Der f*-pulsed, nontreated DCs and challenged with *Der f* contained higher numbers (*P* < .05) of both eosinophils and lymphocytes as compared with control mice. The data in Figure 3 also clearly indicates that treatment of *Der f*-pulsed DCs with EP in vitro prevented significantly (*P* < .05) increase in eosinophils and lymphocytes in NLF induced by *Der f* intranasal challenge.

### 3.3. Influence of EP on Cytokine Production from DCs In Vivo

**Figure 4:**
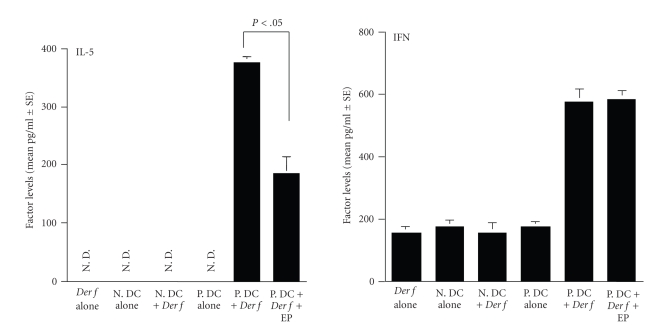
Influence of nasal antigenic challenge on inflammatory cytokine appearance in nasal lavage fluid obtained from mice instilled nasally with *Dermatophagoides farinae* (*Der f *)-pulsed dendritic cells. BALB/c mice were instilled nasally with *Der f*-pulsed and 25 ng/mL epinastine hydrochloride (EP)-treated DCs. Nasal lavage was performed 24 hours after the final *Der f* allergen exposure. The data are expressed as the mean pg/mL ± SE of five mice. This is one of two different experiments, which gave reproducible results. *Der f* alone: *Der f* allergen exposure alone; N. DC alone: nonpulsed DC instilled; N. DC + *Der f*: nonpulsed DC instilled and *Der f* allergen exposure; P. DC alone: *Der f*-pulsed DC instilled; P. DC + *Der f* : *Der f*-pulsed DC and *Der f* allergen exposure; P. DC + *Der f* + EP: *Der f*-pulsed, EP-treated DC and *Der f* exposure. N. D.: not detected.

The third experiments were carried out to examine whether in vitro changes in the ability of DCs to produce cytokines was also observed in vivo. NLF obtained from mice instilled nonpulsed DCs contained much lower levels of IL-5, even when these mice were challenged intranasally with *Der f* (Figure 4). However, intranasal challenge with *Der f* into mice harboring *Der f*-pulsed DCs caused significant increase in IL-5 levels in NLF, and this activity of *Der f*-pulsed DCs to produce IL-5 in vivo was significantly suppressed by the pretreatment with EP in vitro (Figure 4). We then examined the influence of intranasal instillation of DCs pretreated with EP on the appearance of IFN-*γ* in NLF. As shown in Figure 4, nasal instillation of *Der f* into mice harbored *Der f*-pulsed DCs caused significant increase in IFN-*γ* levels in NLF as compared with that from control DCs harboring mice. In vitro treatment of *Der f*-pulsed DCs with EP could not suppress the ability of cells to produce IFN-*γ* in response to intranasal challenge with *Der f*. NLF obtained from mice harboring *Der f*-pulsed DCs and treated EP contained similar levels (not significant; *P* > .05) of IFN-*γ* to that from *Der f*-pulsed and nontreated DCs.

## 4. Discussion

The present study examined the influence of *Der f* and EP on the function of DCs. To do this, we firstly pulsed DCs with *Der f*, and then examined the influence of EP on the production of immunomodulatory cytokines (IL-10 and IL-12) in vitro. Second, these DCs were instilled intranasally into naïve mice, followed by *Der f* exposure, and then cytokine profile and cellular changes in NLF were compared to examine whether in vitro changes in DCs by EP could be actually reflect in vivo.

DCs are the most potent APCs in priming naïve T and B cells, and they play a key role in determining the type of immune response [12–14]. Located in an immature state at the site of antigen entry, DCs actively capture and process antigens, and migrate to the draining lymph nodes, where they present processed peptides to naïve T cells and initiate various immune responses [19]. During this process, DCs produce several types of cytokines, which affect the differentiation of activated T cells into Th1 or Th2 cells [12–14]. The cytokine IL-12 promotes Th1 responses and stimulates activated T cells to produce IFN-*γ*, whereas it inhibits the development of IL-4-producing Th2 cells in response to *D. pteronyssimus* [20]. In addition, IL-12 markedly suppresses the IL-4-induced IgE production from human peripheral blood leukocytes in vitro [21, 22], suggesting that IL-12 might be capable of downregulating allergic immune responses. This hypothesis may be supported by the observation that systemic and intranasal administration of IL-12 into previously sensitized mice could inhibit the airway hyperreactivity against specific antigens and metacholine as well as eosinophilia in bronchoalveolar lavage fluid [23, 24]. It is also supported by the experimental evidence that mucosal gene transfer of IL-12 via a vaccinia virus vector inhibited the ability of lung lymphoid cells to produce IL-4 and IL-5, but not IFN-*γ* and prevented the development of airway hyperreactivity in a mouse model of ovalbumin-induced asthma [25]. IL-10 is first identified at the molecular level as a factor produced by Th2 cells, which inhibited the production of cytokines by Th1 cells. Although produced by Th2 cells, IL-10 inhibits many Th2 functions relevant to allergic disorders. IL-10 also inhibits the function of mast cells and eosinophils, which are associated with allergic responses, and favorably modulates IgE to IgG4 ratios [26]. On the other hand, IL-10 is reported to enhance the formation of Th2 cells by downregulating IL-12 production [27], suggesting that the role of IL-10 on the development of allergic immune responses is controversial. In general, however, at least at the DC level, it is likely that DCs induce a Th1 response in high IL-12 and low IL-10, whereas they induce a Th2 response in low IL-12 and high IL-10 [28, 29]. The present results clearly showed that EP at 25.0 ng/mL, which is similar to a therapeutic blood level (26.9 ± 9.1 ng/mL) when the patients were orally given EP at 20 mg [30], exerts both suppressive effect on IL-10 production and enhancive effect on IL-12 production from DCs. Taken together, therefore, it is reasonably to speculate that EP inhibits the development of Th2 immune response through the modulation of the ability of DCs to produce immunomodulatory cytokines, especially IL-10 and IL-12, and results in prevention of propagation of allergic inflammation in nasal walls. This speculation may be supported by the observation that administration of soluble ovalbumin together with a monoclonal antibody against IL-10 receptor to mice led to the enhancement of a Th1 response upon rechallenge [31].

In addition to IL-10 and IL-12, DCs are well known to be able to produce several types of proinflammatory cytokines. In the present study, we demonstrated that treatment with EP inhibits the ability of DCs to produce cytokines such as IL-6 and TNF-*α*. IL-6 is well known to be a multifunctional cytokine that can promote the development of inflammatory responses. In addition to playing essential roles in the process of growth and differentiation of inflammatory cells such as mast cells and B cells [32], this cytokine displays other biological properties; IL-6 is the major inducer of C-reactive protein (CRP) production in the liver, which can enhance the ability of macrophages and neutrophils to produce inflammatory mediators in local inflammatory lesions [33, 34]. TNF-*α* is also well accepted to exert several specific immunomodulatory effects [35, 36]. Furthermore, these two cytokines and CRP are reported to be able to enhance the activity of inflammatory cells to produce free radicals, including nitric oxide and superoxide radicals, which are the most important final effector molecules of inflammatory diseases [36]. Together with these reports, the present results may suggest that the suppressive effect of EP on cytokine production from DCs may underlie the therapeutic mode of action of EP on allergic diseases such as allergic rhinitis and atopic dermatitis.

Before obtaining the conclusion that the suppressive activity of antihistamine, especially EP on Dc functions contributes, at least in part, to the therapeutic mode of action of the agent, it is essential for examining whether the changes of DC functions in vitro could alter the nasal inflammatory responses against specific allergic challenge in vivo. Therefore, we then instilled nasally with *Der f*-pulsed DCs treated with EP into naïve mice, and examined the degree of allergic inflammatory responses in the nasal wall against *Der f* challenge. The present results of in vivo experiments were consistent with those in vitro experiments and demonstrated that mice instilled with *Der f*-pulsed DCs developed allergic inflammation in nasal wall as assessed by eosinophil appearance in NLF, which concomitant with increased production of IL-5. In contrast, mice instilled with *Der f*-pulsed DCs treated with EP showed a reduction in nasal eosinophil count and IL-5 production, but increased IFN-*γ*, indicating that the in vitro functional changes noted in *Der f*-pulsed DCs by the treatment with EP in vitro were supported by these in vivo experiments. The mechanisms by which instillation of *Der f*-pulsed DCs pretreated with EP exerts the suppressive effect on Th2 type cytokine, IL-5 production in vivo are not clear at present. Our previous work clearly showed that EP at 25 ng/mL significantly inhibits the expression of costimulatory molecules, CD40, CD80, and CD86, which are essential for the development of T cell-dependent immune responses, on human peripheral blood monocyte-derived DCs (MoDCs) in vitro [37]. It is also reported that EP could decrease the ability of MoDCs to both stimulate CD4^+^ T cells and phagocyte antigens in vitro [37], suggesting that these inhibitory action of EP may be responsible for the modulation of appearance of T-cell cytokines, IL-5, and IFN-*γ* in NLF after *Der f* stimulation.

Although the present results strongly indicate that antihistamine, especially EP, inhibits DCs-induced Th2 skewed immune responses and results in favorable modification in DCs-induced allergic immune responses in nasal walls, the mechanism(s) by which EP could modulate DC functions induced by specific allergen stimulation are not clear at present. Several endogenous mediators have been reported that modulate DC functions, including adenosine [38], phosphodiesterase [39], prostaglandins [40], and cAMP [41]. Among these, the influence of cAMP on cytokine production from DCs was extensively studied and reported that the increase in cAMP activation caused suppression of IL-12 production and enhanced the ability of cells to produce IL-10 [39]. It is also reported that rolipram and ciaprost, which are pharmacological agents led to cAMP activation, enhanced IL-10 production from human peripheral blood monocytes in response to LPS stimulation [42]. Judging from these reports, the present results may be interpreted that treatment of DCs with EP as well as azelastine hydrochloride, an H_1_ receptor antagonist [43], inhibited cAMP activation, which was enhanced by *Der f* stimulation, and results in changes in the ability of DCs to produce immunomodulatory cytokines. There is growing evidence that cAMP response element-binding protein (CREB), which is a 48-*κ*Da nuclear transcription factor bound to cAMP response element, modulates the ability of cells to produce proinflammatory cytokines, such as TNF-*α* and IL-6. Inhibition of CREB is reported to decrease IL-6 mRNA expression in vascular smooth muscle cells and peritoneal mouse macrophages in response to the stimulation with thrombin [44] and deoxynivalenol [45], respectively. It is also reported that inhibition of CREB activation suppress the ability of macrophage cell line, RAW264.7, to produce TNF-*α* in response to LPS in vitro [46, 47], suggesting that EP inhibits IL-6 and TNF-*α* production from DCs induced by specific allergen stimulation through the suppression of CREB activation in vitro. This speculation may be supported by the observation that terfenadine, an H_1_ receptor antagonist, exerts the suppressive effect on CREB activation in mouse cholangiocytes in vitro [48]. Anyway, further experiments are required to clarify the molecular mechanisms by which EP could modulate DC functions, especially cytokine production.
